# Development, optimization, and preliminary evaluation of a novel artificial intelligence tool to promote patient health literacy in radiology reports: The Rads-Lit tool

**DOI:** 10.1371/journal.pone.0331368

**Published:** 2025-09-03

**Authors:** Rushabh H. Doshi, Kanhai Amin, Shin Mei Chan, Manroop Kaur, Simar S. Bajaj, Pavan Khosla, Veer T. Kothari, Ali Mozayan, Irena Tocino, Sophie Chheang

**Affiliations:** 1 Yale School of Medicine, New Haven, Connecticut, United States of America; 2 Yale College, New Haven, Connecticut, United States of America; 3 UCSF Department of Radiology & Biomedical Imaging, San Francisco, California, United States of America; 4 Department of Radiology and Biomedical Imaging, Yale School of Medicine, New Haven, Connecticut, United States of America; 5 Harvard College, Cambridge, Massachusetts, United States of America; Makere University College of Health Sciences, UGANDA

## Abstract

Radiology reports are an integral part of patient medical records; however, these reports often contain complex medical terminology that are difficult for patients to comprehend, potentially leading to anxiety, misunderstanding, and misinterpretation. The development of user-friendly instruments to improve understanding is thus critically important to enhance health literacy and empower patients. In this study, we introduce a novel artificial intelligence (AI) interface, the Rads-Lit Tool, which can simplify radiology reports for patients using natural language processing (NLP) techniques. This manuscript presents the development process, methodology, and results of the Rads-Lit Tool, demonstrating its potential to simplify radiology reports across various examination types and complexity levels. Our findings highlight that patient-facing AI-driven tools can enhance patient health literacy and foster improved patient-provider communication in radiology.

## 1. Introduction

A cornerstone of modern medicine, imaging has revolutionized diagnosis, treatment planning, and disease monitoring with radiology reports an indispensable component of patient medical records. Traditionally, these reports have been accessible only to radiologists and the referring providers, who then interpret findings for patients [1]. However, the rise of electronic portals has led to an increasing number of patients directly accessing their medical information, with the 21st Century Cures Act mandating access to all parts of the electronic health record (EHR) [2]. While this shift empowers patients to play a more active role in their care, the complexity of medical jargon and acronyms in radiology reports often leads to significant confusion, anxiety, and potential misinterpretation [3,4]. Simply looking up individual terms often fails to provide a cohesive understanding of the report’s narrative and its implications. This underscores a critical need for tools that can translate the entire report findings into plain language, thereby enhancing patient comprehension and facilitating more meaningful patient-provider communication Moreover, advanced imaging modalities continue to evolve rapidly, reinforcing the need for clear, transparent patient-facing reports.

Correspondingly, there has been growing attention toward health literacy and its associations with patient engagement, treatment adherence, and care disparities [5]. Advanced natural language processing (NLP), in turn, has emerged as one promising tool to help bridge the gap between complex medical information and ease of understanding [6–8]. In radiology in particular, NLP techniques have been used in everything from observation detection for diagnostic surveillance to quality assessment to clinical support services [9,10]. A few studies have sought to use NLP to simplify radiology reports, namely through linking terms to the consumer health vocabulary system [11] and the French lexical network [12].

With the rise of large language models (LLMs), OpenAI’s ChatGPT, Google Bard, and Microsoft Bing have also been explored for simplifying radiology reports [13,14]. The main advantage of these tools is their accessibility and comprehensiveness — freely available to anyone with an internet connection and able to simplify an entire radiology report, rather than simply appending a summary, linking to a glossary, or altering the structure. However, there are limitations. While general-purpose LLMs like ChatGPT are accessible, their effectiveness for specialized tasks like simplifying radiology reports heavily depends on user-crafted prompts, which can lead to variable quality and reliability [15,16]. Patients may not possess the expertise to formulate optimal prompts. Rads-Lit addresses this by embedding a systematically optimized prompt within a user-friendly interface, specifically designed for radiology reports. This aims to provide more consistent, reliable, and appropriately simplified outputs compared to ad-hoc use of general LLMs, thereby offering a more dependable solution for enhancing patient health literacy in this domain.

We have used a variety of prompts to assess these LLMs for accuracy and fidelity in simplifying radiology reports [15,16]; however, we have this data only for specific prompts and remain unsure if the LLMs would be inaccurate or inadequate if asked different prompts. Given the infinite possibility of prompts and the corresponding variable quality of responses, patients may not be able to take full advantage of these chatbots to improve their own health literacy. While general LLMs show promise, their direct application by patients for simplifying complex medical texts like radiology reports is fraught with challenges, including prompt variability and inconsistent output quality [13–16]. This highlights a critical gap: the need for specialized, optimized tools that can reliably simplify radiology reports to an appropriate health literacy level while maintaining clinical accuracy. Therefore, this study aimed to address the following research questions:

1.) Can a systematic prompt engineering process identify an optimal LLM prompt to simplify radiology reports to a target 5th-7th grade reading level?

2.) How does a specialized tool (Rads-Lit), utilizing an optimized prompt, perform in terms of readability improvement across various imaging modalities compared to original reports and a basic simplification prompt?

3.) What is the accuracy, completeness, and perceived safety (by radiologists) of the simplified reports generated by such a tool?

We present the development, optimization, and preliminary evaluation of the Rads-Lit Tool, an AI interface designed to address these questions. This work includes a novel methodology for LLM prompt assessment for health literacy, aiming to empower patients with understandable medical information, thereby fostering improved patient-provider communication and informed decision-making.“

## 2. Methods

### 2.1. Development of interface

We developed a proof-of-concept user interface for patients to input their radiology findings and receive a simplified version of their findings (http://radiologyliteracy.org/), utilizing OpenAI’s Davinci application programming interface (API). We have undergone an optimization process, as detailed below, to simplify patients’ imaging report at a reading level recommended by the American Medical Association and National Institutes of Health while maintaining accuracy [17,18].

### 2.2. Dataset selection and modification

We sourced a random selection of 750 radiology reports across diverse examination types (150 MRI, CT, US [ultrasound], X-ray, and Mammogram each) from the MIMIC-III database, a comprehensive dataset available from Beth Israel Deaconess Medical Center [19,20]. (13, 19, 20, 25) A random sub-selection of 25 reports was initially chosen to test our prompts. Redacted physician names in the reports were changed to “Dr. Smith” and redacted dates were changed to “prior.” As this study used only de-identified, publicly available data, our specific analysis was deemed exempt from further institutional IRB review given our institution’s IRB involves analysis of publicly available de-identified data. Given the de-identified nature of the data, patient consent for this specific retrospective study was waived.

### 2.3. Readability scores

To assess readability, we used the validated Gunning Fog (GF), Flesch-Kincaid Grade Level (FK), Automated Readability Index (ARI), and Coleman-Liau (CL) indices, as previous studies have done [16,21–23]. Each of these reading indices output a value related to a reading grade level (RGL) (i.e., output of 7 represents the 7^th^ grade reading level.) Further, in line with previous studies, we averaged the scores across all 4 indices to get an average RGL, aRGL [24].

### 2.4. Prompt engineering and optimization

**Fig 1:**
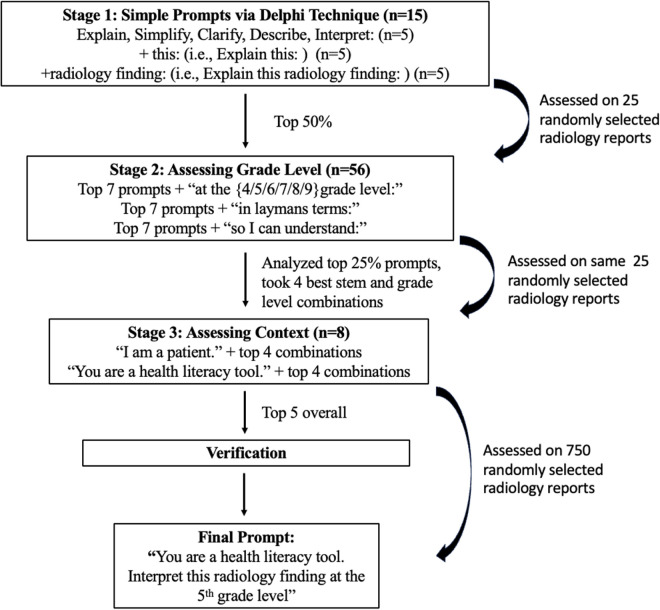
Prompt optimization process.

Our prompt engineering involved a multi-stage iterative process (Fig 1). Stage 1 began with identifying five core simplifying stems (‘simplify’, ‘explain’, etc.) via a Delphi technique, tested with two modifiers for 15 initial prompts. Stage 2 focused on prompts that achieved below the median Stage 1 aRGL, adding grade level specifications and contextual phrases (e.g., ‘so I can understand’), resulting in 56 further prompts. Stage 3 took the top four stem/grade combinations and added two persona-based contexts (‘I am a patient,’ ‘you are a health literacy tool’) for 8 additional prompts. The full list of 79 prompts is detailed in S1 Fig. The five best-performing prompts (lowest median aRGLs) from these stages were then extensively tested on 750 reports, alongside a basic ‘simplify’ prompt, to select the final prompt for the Rads-Lit tool, again using a Delphi method for the final choice based on readability, fidelity and accuracy.

### 2.5. Accuracy, completeness, and comprehension of Rads-lit tool

After selecting the best-performing prompt, three radiologists—2 attendings and 1 resident—assessed 62 of these reports and the corresponding simplified. Specifically, these radiologists evaluated the output for accuracy, completeness, and extraneous information using single-item Likert scales, ranging from 1 (Strongly Disagree, 0–20% agreement) to 5 (Strongly Agree, 80–100% agreement).

### 2.6. Statistical analysis

The non-parametric Wilcoxon signed-rank and rank-sum tests were used for statistical analysis.

## 3. Results

The prompt optimization process is summarized in Fig 1 and Table 1.

**Table 1:** Prompt optimization process and corresponding average readability grade level (aRGL) scores.

Stage	Prompt	Round 1	Average Reading Grade Level	Round 2	Average Reading Grade Level	Round 3	Average Reading Grade Level
1	Explain. ----	X	12.3277				
	Simplify. ----		17.0265				
	Clarify. ----		14.0026				
	Describe. ----	X	13.3636				
	Interpret. ----		14.2223				
	Explain this. ----		15.5284				
	Simplify this. ----		15.4535				
	Clarify this. ----	X	11.8043				
	Describe this. ----	X	13.0073				
	Interpret this. ----		14.1201				
	Explain this radiology finding. ----	X	13.6338				
	Simplify this radiology finding. ----		17.875				
	Clarify this radiology finding. ----	X	13.6909				
	Describe this radiology finding. ----		14.3011				
	Interpret this radiology finding. ----	X	13.9849				
2	Explain this radiology finding so I can understand. ----				12.9838		
	Explain this radiology finding in layman terms. ----				11.7333		
	Explain this radiology finding at a 9th grade level. ----				9.1195		
	Explain this radiology finding at a 8th grade level. ----				10.0755		
	Explain this radiology finding at a 7th grade level. ----				8.6691		
	Explain this radiology finding at a 6th grade level. ----				9.0847		
	Explain this radiology finding at a 5th grade level. ----				9.3682		
	Explain this radiology finding at a 4th grade level. ----				9.323		
	Clarify this radiology finding so I can understand. ----				14.8914		
	Clarify this radiology finding in layman terms. ----				10.3825		
	Clarify this radiology finding at a 9th grade level. ----				10.5219		
	Clarify this radiology finding at a 8th grade level. ----				10.2292		
	Clarify this radiology finding at a 7th grade level. ----				9.4175		
	Clarify this radiology finding at a 6th grade level. ----				9.379		
	Clarify this radiology finding at a 5th grade level. ----				9.264		
	Clarify this radiology finding at a 4th grade level. ----				8.2593		
	Interpret this radiology finding so I can understand. ----				14.3113		
	Interpret this radiology finding in layman terms. ----				12.6786		
	Interpret this radiology finding at a 9th grade level. ----				11.0223		
	Interpret this radiology finding at a 8th grade level. ----				7.9129		
	Interpret this radiology finding at a 7th grade level. ----				9.2156		
	Interpret this radiology finding at a 6th grade level. ----				8.4567		
	Interpret this radiology finding at a 5th grade level. ----			X	7.5413	X	
	Interpret this radiology finding at a 4th grade level. ----			X	6.805	X	
	Explain so I can understand. ----				11.1571		
	Explain in layman terms. ----				11.7876		
	Explain at a 9th grade level. ----				10.3177		
	Explain at a 8th grade level. ----				12.2255		
	Explain at a 7th grade level. ----				10.396		
	Explain at a 6th grade level. ----				11.369		
	Explain at a 5th grade level. ----				12.1338		
	Explain at a 4th grade level. ----				10.124		
	Describe so I can understand. ----				15.0518		
	Describe in layman terms. ----				13.3335		
	Describe at a 9th grade level. ----				9.7826		
	Describe at a 8th grade level. ----				11.0025		
	Describe at a 7th grade level. ----				10.0836		
	Describe at a 6th grade level. ----				11.4504		
	Describe at a 5th grade level. ----				9.8969		
	Describe at a 4th grade level. ----				8.6372		
	Clarify this so I can understand. ----				11.8043		
	Clarify this in layman terms. ----				12.4277		
	Clarify this at a 9th grade level. ----				10.425		
	Clarify this at a 8th grade level. ----				9.6958		
	Clarify this at a 7th grade level. ----				9.7717		
	Clarify this at a 6th grade level. ----				10.481		
	Clarify this at a 5th grade level. ----				9.8041		
	Clarify this at a 4th grade level. ----				8.1064		
	Describe this so I can understand. ----				14.308		
	Describe this in layman terms. ----				10.1574		
	Describe this at a 9th grade level. ----				9.4075		
	Describe this at a 8th grade level. ----				9.8597		
	Describe this at a 7th grade level. ----				8.0875		
	Describe this at a 6th grade level. ----				9.2578		
	Describe this at a 5th grade level. ----			X	8.4589		
	Describe this at a 4th grade level. ----			X	7.3842	X	
3	I am a patient. Interpret this radiology finding at a 5th grade level. ----						9.7724
	You are a health literacy tool. Interpret this radiology finding at the 5th grade level. ----					X	7.3258
	I am a patient. Interpret this radiology finding at a 4th grade level. ----						8.8212
	You are a health literacy tool. Interpret this radiology finding at the 4th grade level. ----					X	7.7558
	I am a patient. Describe this at a 5th grade level. ----						12.3456
	You are a health literacy tool. Describe this at a 5th grade level. ----						10.3601
	I am a patient. Describe this at a 4th grade level. ----						9.423
	You are a health literacy tool. Describe this at a 4th grade level. ----						9.5407

After determining the 5 best-performing prompts in the pilot analysis across Stages 1–3 (Table 1), we re-calculated the average readability scores of these prompts across 750 radiology reports (Table 2). These prompts, namely Prompts A-E, demonstrated significantly improved readability scores compared to the original radiology report and the common prompt, “Simplify:” across all four readability indexes tested (p < 0.0001) (Table 1, S2 Fig). The final five prompts had comparable, reading score differences, with aRGLs of 6.1 to 6.3, and were not statistically significant.

**Table 2:** Average readability grade level (aRGL) scores of the original radiology report, and outputs from the basic prompt “simplify” and the 5 best prompts. *Average represents aRGL. Medians and Quartile 1 – Quartile 3 are depicted for each prompt tested on 255 radiology reports. Prompt A-E are significantly lower than both the Report and Simplify for all scales, p < 0.0001. Simplify is significantly lower than the Report for all modalities, p < 0.0001.*

	Overall	CT	Mammogram	MRI	Ultrasound	X-ray
Raw Report	13.7 (11.9-15.7)	13.9 (12.0-15.9)	13.2 (11.7-15.5)	13.7 (11.7-15.5)	13.6 (11.2-16.4)	14.1 (12.1-15.8)
Simplify	11.3 (9.5-13.4)	11.3 (9.4-14.3)	10.8 (9.2-12.2)	11.8 (10.1-13.0)	11.3 (9.3-13.5)	11.8 (9.8-14.2)
Prompt A	6.3 (5.2-7.4)	6.3 (5.3-7.6)	6.4 (5.4-7.5)	6.3 (5.4-7.2)	6.0 (4.6-7.6)	6.2 (5.0-7.3)
Prompt B	6.1 (4.9-7.3)	6.2 (4.9-7.2)	6.4 (5.0-7.6)	6.1 (5.1-7.1)	5.9 (4.5-7.2)	6.0 (4.7-7.5)
Prompt C	6.0 (4.8-7.1)	6.0 (4.7-7.1)	6.2 (5.1-7.5)	5.9 (4.9-6.8)	5.6 (4.4-6.9)	6.2 (4.8-7.8)
Prompt D	6.2 (5.2-7.5)	6.3 (5.5-7.4)	5.9 (5.2-7.1)	6.4 (5.6-7.7)	6.2 (4.9-7.6)	6.3 (4.7-7.4)
Prompt E	6.1 (4.9-7.3)	6.1 (5.1-7.3)	6.3 (5.0-7.2)	6.2 (5.2-7.3)	6.0 (4.7-7.4)	5.8 (4.6-7.1)

Given that were no statistical differences between the five prompts, we utilized the Delphi method to decide our prompt. 2 reviewers blindly assessed 5 outputs from each of the 5 prompt on fidelity and accuracy and chose prompt D, “You are a health literacy tool. Interpret this radiology finding at the 5^th^ grade level:”. Despite not having the lowest readability score, we chose this prompt because it best retained fidelity while clearly defined the role of the LLM and provided a goal grade level (Table 2).

**Fig 2:**
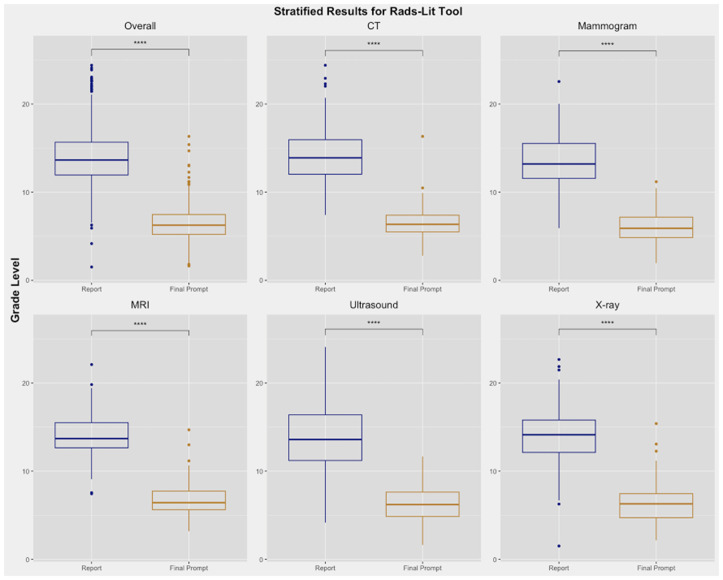
Stratification between included modalities for the main primary prompt: Prompt D. *, **, ***, **** correspond to p < 0.05, p < 0.01, p < 0.001, and p < 0.0001, respectively. aRGLs of the the final prompt and report were used.

In simplifying 750 radiology reports, our selected prompt for our tool (utilizing Prompt D) shows that all four readability indices tested showed statistically significant improvement in readability scores of findings compared to raw radiologist reports (N = 750, p < 0.0001) (Table 1, Fig 2, S3 Fig). The chosen prompt for our interface produced output with a median of 55 [38–72.5] words.

Across all imaging types, our tool (utilizing prompt D) simplified radiology reports from an aRGL of 13.7 to an aRGL of 6.2 (Fig 2). CT radiology reports had an aRGL of 13.9, and our tool provided simplified outputs with an aRGL of 6.3. This simplification held across different examination types, including US (13.6 to 6.2), Mammogram (13.2 to 5.9), MRI (13.7 to 6.4), and X-ray (14.1 to 6.3; Table 1).

**Fig 3:**
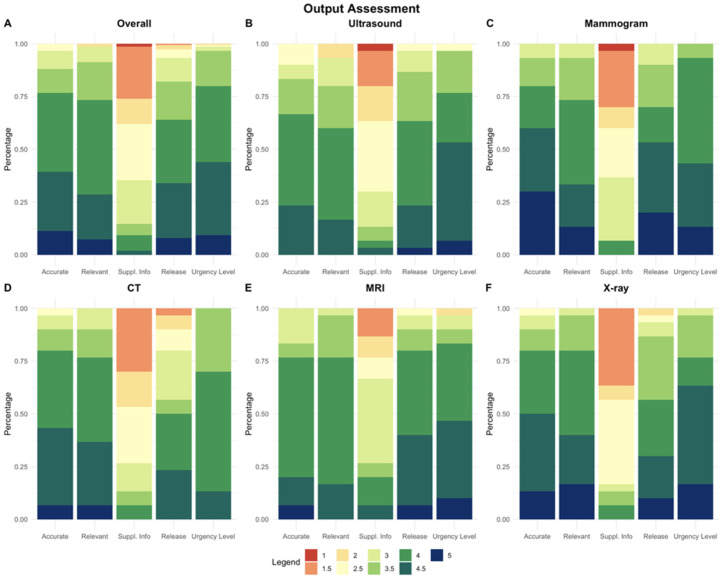
Accuracy, completeness, and inclusion of extraneous information of Rads-Lit tool.

With the optimized prompt, we assessed our tool for accuracy, completeness of information, inclusion of beneficial supplementary information not found, appropriate urgency, and comfort providing output without supervision using a 5-level likert-type evaluation (Fig 3). For accuracy of the information, both radiologists predominantly found most simplified reports to be free of inaccuracies or misleading details, with combined agreement or strong agreement recorded 255 times out of 300 outputs (85%). However, the radiologists indicated disagreement or strong disagreement with this sentiment for 24 outputs (8%). Regarding the inclusion of all pertinent or actionable details from the original reports in the simplified versions, 249 outputs (83%) conveyed agreement or strong agreement. They expressed disagreement or strong disagreement with 18 (6%) outputs. The provision of beneficial supplementary information in the simplified reports not found in the original impression was found in 81 outputs (27%), while 189 outputs (63%) disagreed or strongly disagreed. On the matter of comfortably sharing the simplified reports directly with patients without additional oversight, the radiologists felt expressed agreement or strong agreement to comfortably sharing reports without additional supervision in 230 outputs (76.7%), while they expressed reservations for 39 outputs (13%). The radiologists felt, in 261 of the outputs (87%), that the simplified reports adeptly communicated the required urgency. Disagreement or strong disagreement was recorded in only 8 instances (2.7%).

## 4. Discussion

Our proof-of-concept study presents the development and evaluation of the Rads-Lit patient interface. Through an iterative comparative assessment, we evaluated 79 distinct prompts to determine which five best simplified radiologist reports, achieving the lowest RGLs, relative to the common prompt (“Simplify”). Ultimately, we selected the prompt: “You are a health literacy tool. Interpret this radiology finding at the 5^th^ grade level” for further testing based on our team’s previous study on the importance of context for OpenAI’s LLM [25]. Evaluations by 2 radiologists confirmed that the output from this prompt was accurate 85% of the time with little to no extraneous content relative to the original radiology report. While our findings suggest there is room for model refinement, they also underscore the importance of balancing the clarity of simplified reports with preserving nuanced clinical details integral to patient care. Importantly, simplified language does not equate to clinical guidance. Without proper framing, patients may misinterpret these outputs as standalone diagnostic conclusions.

Given the critical need to promote radiological literacy, approaches such as summary statements, [26] language glossaries, [27] structured templates with standardized lexicon [28,29], video radiology reports [30,31], and listing the radiologist’s phone number have been proposed [32]. This study highlights the utility of using NLP and LLMs to simplify radiology reports, as well as several areas of further research. For example, radiologists’ opinions diverged regarding the inclusion of supplementary information, with only 27% of the outputs found beneficial and a notable 63% disagreement, suggesting that a one-size-fits-all approach may not be ideal. This variability reinforces the need for clear communication that simplified reports are meant to enhance, rather than replace clinical consultation. Some patients might benefit from additional context, while others might find this supplementary information extraneous or confusing. Additionally, the collective comfort of the radiologists in sharing 76.7% of the simplified reports directly with patients indicates the tool’s potential in promoting patient autonomy and comprehension. Still, given the novelty of this technology and risk of hallucinations [33,34], it is unlikely that radiologists would trust the Rads-Lit patient interface to operate autonomously. Indeed, in a previous single-center survey, 76.9% of radiologists reported that they would not support AI-generated simplifications of reports without a manual check [35]. A safety net, possibly in the form of a reviewing radiologist, will likely still be necessary. Hospitals and health systems must consider embedding controls within such tools, such as requiring clinician review before release, clear disclaimers on patient-facing outputs, and integration with the electronic health record to ensure traceability. These institutional guardrails are critical to prevent misuse, including inappropriate self-management or misinformed clinical decision-making by patients.

Patients are already using LLMs like OpenAI’s ChatGPT, Google Bard, and Microsoft Bing to better understand their medical care; however, given the infinite possibility of prompts, most people are not providing additional context that they are a patient, that the chatbot should act as a health literacy tool, or that simplification should happen at a specific grade level [36–38]. Indeed, our previous work has shown that the prompt “Simplify” on ChatGPT-3.5 consistently produces excessively complicated outputs for the average American’s 7^th^ grade reading level [13,17,18]. Other research from our group suggests that the level of simplification differs based on racial context: Open AI’s ChatGPT-3.5 and ChatGPT-4 simplified radiology reports at a higher reading level for those who self-identified as White or Asian, when compared to those who self-identified as Black or American Indian/Alaska Native [39]. These findings raise important concerns about equity and implicit bias in LLM-generated outputs. While the reasons for these disparities remain unclear, they may reflect systemic inequities embedded in the training data or variation in how different demographic identifiers interact with the model [40].

Some small studies have explored the accuracy of LLM-simplified radiology reports with variable results [41]. For example, using 3 fictitious radiology reports, Jeblick et al. created 15 simplified reports and found that one-third of these reports had incorrect statements, missing key medical information, or potentially misleading passages [42]. Tepe et al. analyzed 30 simplified radiology reports and found that their readability and understandability was significantly improved, although their accuracy in assessing the urgency of medical conditions was inadequate [43]. For contrast, analysis of simplified reports from 20 cardiovascular MRIs and 60 shoulder, knee, and lumbar spine MRIs found that GPT-4 produced highly accurate reports with minimal confusing or inaccurate output [44,45]. Our group has similarly shown that, in analysis of 150 mammography, X-ray, CT, MRI, and ultrasound scans, radiologists found that 83–86% of radiology reports simplified by GPT 3.5 and 4 had no errors and all essential information [16]. However, to our knowledge, there has been no previous work that has comprehensively assessed the accuracy, completeness of information, appropriate urgency, and comfort providing output without supervision, especially in such a large sample size with 750 radiology reports across diverse examination types. Our results suggest the import of implementing standard prompts and guidelines for LLM-based patient education in order to maximize the utility of these tools and to improve equity in health communication.

Our proof-of-concept tool explores the capabilities of LLMs and suggests that these tools can be safely incorporated into radiology practice. With a median count of 55 words in this study, a radiologist should be able to review the simplified output quickly, but the benefits to patients must be weighed against these disruptions to providers, as RVUs may not accommodate for this additional review time and a separate billing code is unlikely. An example of an implementation of such a technology is in a speech recognition and reporting platform, where LLMs and NLP could readily generate a simplified summary, which the radiologist can proof, and if needed, edit. Or, this technology could be implemented in EHRs allowing a radiologist to review simplified output before signing a report. There is evidence a simplified report or summary may benefit patients and improve patient satisfaction scores, but adoption by providers and impact on providers must also be assessed. Importantly, we must address concerns about the unintended consequences. What if, for instance, these simplified reports lead to heightened anxiety? And does it truly offer time-saving benefits to referring physicians when they discuss findings with their patients? Although our work is foundational, it’s essential to be proactive in our discussion and think about the potential implications [46].

This study is not without its limitations. For one, we relied primarily on readability metrics to guide our prompt engineering. Although we utilized the Delphi method to look at the outputs of the 5 prompts with the lowest readability scores to identify the best prompt and ensure that the prompts retained fidelity and accuracy, we did not use accuracy to identify our prompt until that point. Second, the readability metrics used in this study are language and structure-focused, so these measures may not necessarily capture comprehensibility from a medical perspective. Additionally, readability metrics may not adequately capture patient literacy needs. This highlights the need for further research to refine the interface and ensure its applicability across diverse patient populations and radiology subspecialties, as well as explore the potential for personalized approaches to simplifying radiology reports, considering individual patient characteristics, such as age, education, and prior medical knowledge. Moreover, future iterations must prioritize clear guardrails and user education to ensure that patients understand simplified reports are adjunctive, not directive, and that clinical follow-up remains essential. Finally, we have utilized 2 attendings and a resident with the likert scale to assess these prompts when in reality the incorporation of patient feedback and physician input on the simplified reports is crucial to evaluate the tool’s real-world usability and its potential for enhancing patient-provider communication. Future multi-site studies would validate these readability metrics in real-world settings, ensuring their reliability and relevance to patient outcomes.

It is crucial to reiterate that the Rads-Lit Tool is designed as a health literacy aid to simplify existing radiology report text, not as a diagnostic or clinical decision support system. As rightly noted by challenges in the broader AI field, these models, including the one underpinning Rads-Lit, currently lack the ‘Gestalt’ understanding required to interpret findings within the full, complex clinical context of an individual patient, especially concerning comorbidities or the relative intensity of multiple pathologies. While the tool improves access to comprehension, it does not obviate the need for follow-up with a referring clinician. Misinterpretation, such as assuming a non-urgent finding requires no action or self-treating based on the simplified language, poses real risks if safeguards are not in place. Our tool does not attempt this; its purpose is solely to make the concluded report more comprehensible to patients after it has been finalized by a radiologist.

**Fig 4:**
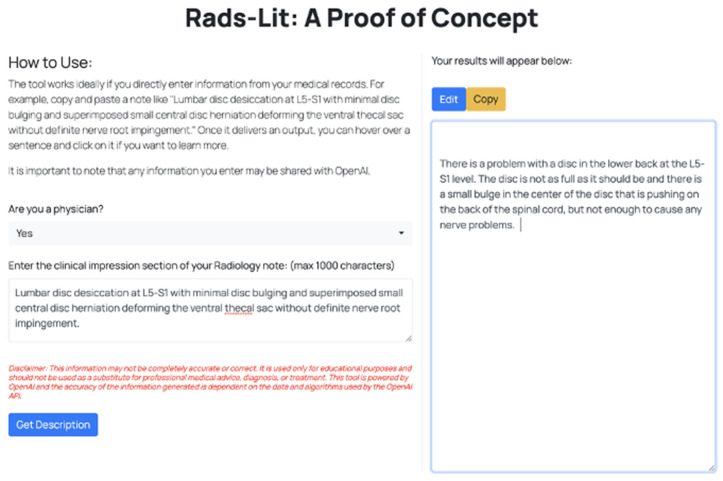
Rads-lit interface.

We believe that this proof-of-concept tool can help begin a discussion of how to utilize such LLMs for patient-centered care in radiology. In our tool, we also showcase preliminary features (that remain untested) to allow patients to press “explain more” if they don’t understand or want to learn more about a particular sentence of the generated output and allow physicians to edit responses if they are using the tool to provide patients a better understanding of their report findings (Fig 4). As LLM solutions become more common, discussion regarding the implementation of such tools is of utmost importance.

In conclusion, this study outlines the development and preliminary evaluation of Rads-Lit, demonstrating that an AI tool with optimized prompting can significantly improve the readability of radiology reports. Our findings suggest its potential as a specialty-specific health literacy aid, shifting the onus of prompt engineering from the patient to a systematically designed interface (Fig 4). While improved patient-provider communication and patient-centered care are key goals, this study represents an initial step. Extensive further research, including direct patient feedback, validation across diverse populations and clinical settings, and assessment of real-world impact on patient understanding and outcomes, is crucial before such tools can be broadly incorporated into clinical practice [47]. Most importantly, any implementation of such tools must prioritize patient safety by incorporating robust safeguards to prevent misinterpretation and inappropriate self-management, ensuring that simplified reports enhance rather than replace essential clinical relationships. Continued collaboration among patients, clinicians, developers, and policymakers will be essential to responsibly harness this technology to support, rather than replace, human-centered care.

## Supporting information

S1 Fig All prompts tested are depicted. Best prompts after each stage are denoted.
(PNG)

S2 Fig 5 best prompts compared to a basic prompt “simplify. ” *, **, ***, **** correspond to p < 0.05, p < 0.01, p < 0.001, and p < 0.0001, respectively. Dashed line depicts 8^th^ grade level.
(PNG)

S3 Fig Rads lit vs radiologist report. *, **, ***, **** correspond to p < 0.05, p < 0.01, p < 0.001, and p < 0.0001, respectively.
(PNG)

S1 Data Data Plos. (XLSX)
