# Supplementary figures and images for: Development, optimization, and preliminary evaluation of a novel artificial intelligence tool to promote patient health literacy in radiology reports: The Rads-Lit tool
Source: PLoS One. 2025 Sep 3;20(9):e0331368. doi: 10.1371/journal.pone.0331368 (PMC12407389; doi:10.1371/journal.pone.0331368)

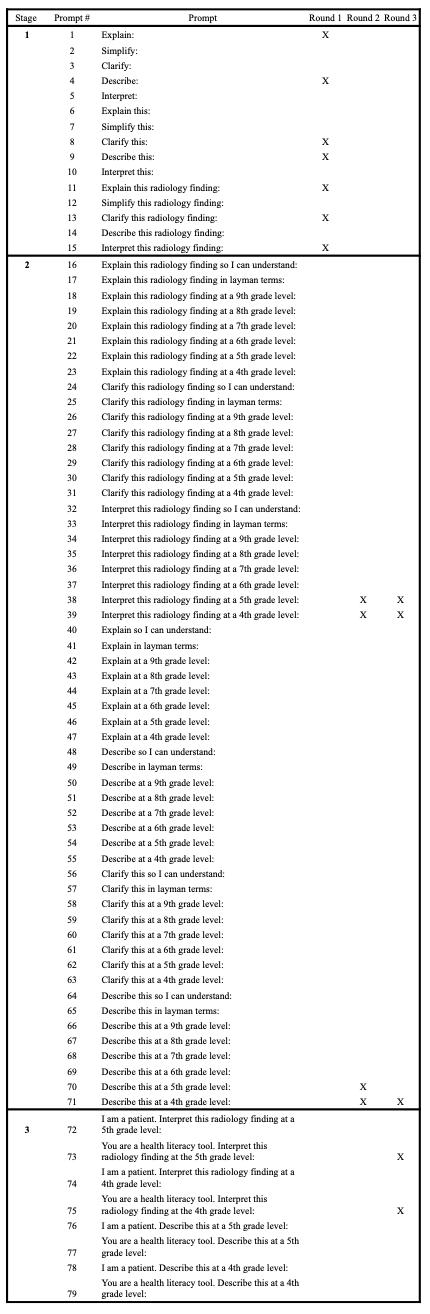

Supplement: S1 Fig — Best prompts after each stage are denoted. (PNG) [file pone.0331368.s001.png]

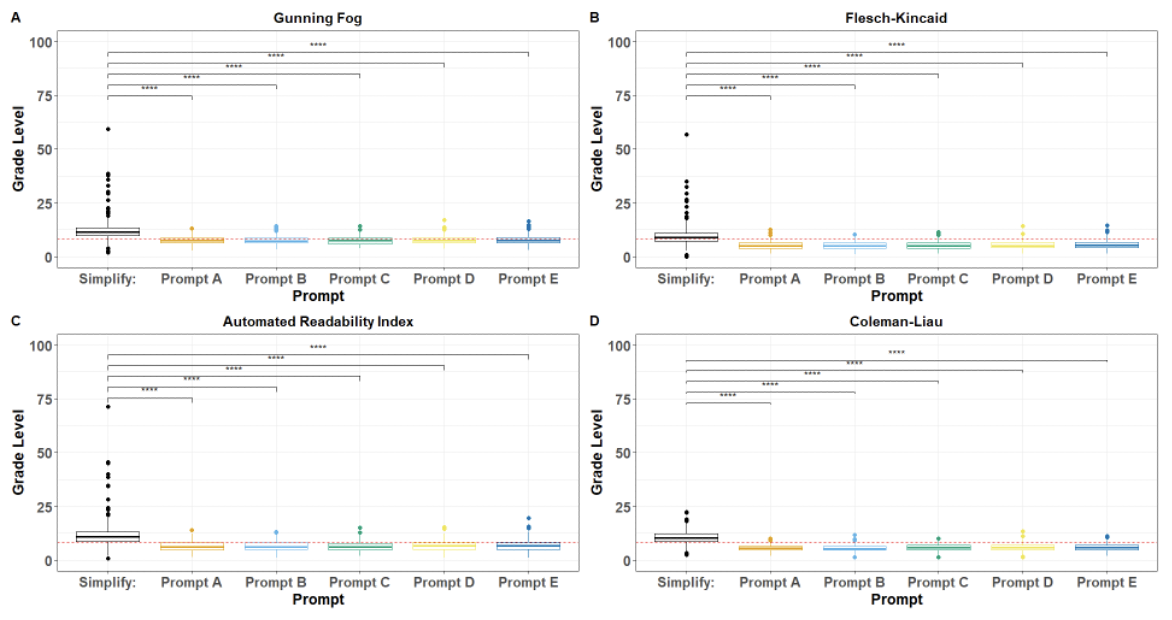

Supplement: S2 Fig — ” *, **, ***, **** correspond to p < 0.05, p < 0.01, p < 0.001, and p < 0.0001, respectively. Dashed line depicts 8th grade level. (PNG) [file pone.0331368.s002.png]

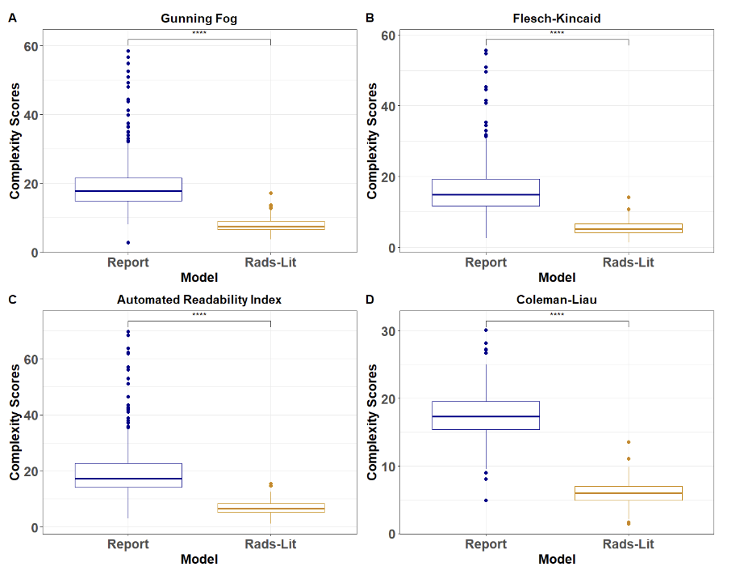

Supplement: S3 Fig — *, **, ***, **** correspond to p < 0.05, p < 0.01, p < 0.001, and p < 0.0001, respectively. (PNG) [file pone.0331368.s003.png]
